# Laparoscopic-assisted disinvagination and polypectomy for multiple intussusceptions induced by small intestinal polyps in patients with Peutz-Jeghers syndrome: a case report

**DOI:** 10.1186/s12957-021-02133-5

**Published:** 2021-01-21

**Authors:** Masaaki Yamamoto, Kazuya Iwamoto, Rei Suzuki, Yosuke Mukai, Tomohira Takeoka, Kei Asukai, Naoki Shinno, Hisashi Hara, Takashi Kanemura, Nozomu Nakai, Shinichiro Hasegawa, Keijiro Sugimura, Naotsugu Haraguchi, Junichi Nishimura, Hiroshi Wada, Hidenori Takahashi, Chu Matsuda, Masayoshi Yasui, Takeshi Omori, Hiroshi Miyata, Masayuki Ohue, Masaru Murata

**Affiliations:** 1 Department of Gastroenterological Surgery, Osaka International Cancer Institute, 3-1-69 Otemae, Chuo-ku, Osaka, 541-8567 Japan; 2 Department of Surgery, JCHO Hoshigaoka Medical Center, 4-8-1, Hoshigaoka, Hirakata, Osaka, 573-8511 Japan; 3 Department of Surgery, Osaka Police Hospital, Kitayama-cho 10-31, Tennozi-ku, Osaka, 543-0035 Japan

**Keywords:** Peutz–Jeghers syndrome, Intussusception, Polyposis, Laparoscopic-assisted polypectomy, Disinvagination

## Abstract

**Background:**

Peutz–Jeghers syndrome (PJS) is a very rare autosomal dominant genetic disorder characterized by hamartomatous polyps in the gastrointestinal tract and hyperpigmentation of the lips, hands, and feet. The hamartomatous polyps in the small intestine often cause intussusception and bleeding.

**Case presentation:**

A 62-year-old male was hospitalized for treatment of deep vein thrombosis and pulmonary embolism. In the small intestine, computed tomography showed three small polyps with intussusceptions. Since the patient had gastrointestinal polyposis and pigmentation of his lips, fingers, and toes, he was diagnosed with PJS. After an inferior vena cava filter was placed, he underwent laparoscopic-assisted surgery. The polyps causing intussusception were resected as far as possible without intestinal resection, since they had caused progressive anemia and might cause intestinal obstruction in the future. The patient was discharged from the hospital on postoperative day 9 without complications.

**Conclusions:**

Laparoscopic-assisted disinvagination and polypectomy is a useful, minimally invasive treatment for multiple intussusceptions caused by small intestinal polyps in patients with PJS.

**Supplementary Information:**

The online version contains supplementary material available at 10.1186/s12957-021-02133-5.

## Background

Peutz–Jeghers syndrome (PJS) is an autosomal dominant genetic disorder characterized by gastrointestinal polyposis and mucocutaneous pigmentation [1]. It was first identified by Peutz in 1921, and Jeghers published a description of the syndrome in 1949 [2–4]. Bruwer et al. named the condition PJS based on the work of Peutz and Jeghers [5]. Its incidence has been estimated to range from 1 in 50,000 to 1 in 200,000 live births [1, 6].

PJS is characterized by germline mutations in the serine–threonine kinase 11 gene (*STK11*), also known as liver kinase B1 [7, 8]. *STK11* is localized on chromosome 19p13.3 and functions as a tumor suppressor gene [9]. The polyps in PJS patients may induce bleeding, small intestinal intussusception, or obstruction, and these conditions usually lead to the initial diagnosis of PJS. We reported the case of a PJS patient with multiple small intestinal intussusceptions who presented due to progressive anemia.

### Case presentation

**Fig. 1:**
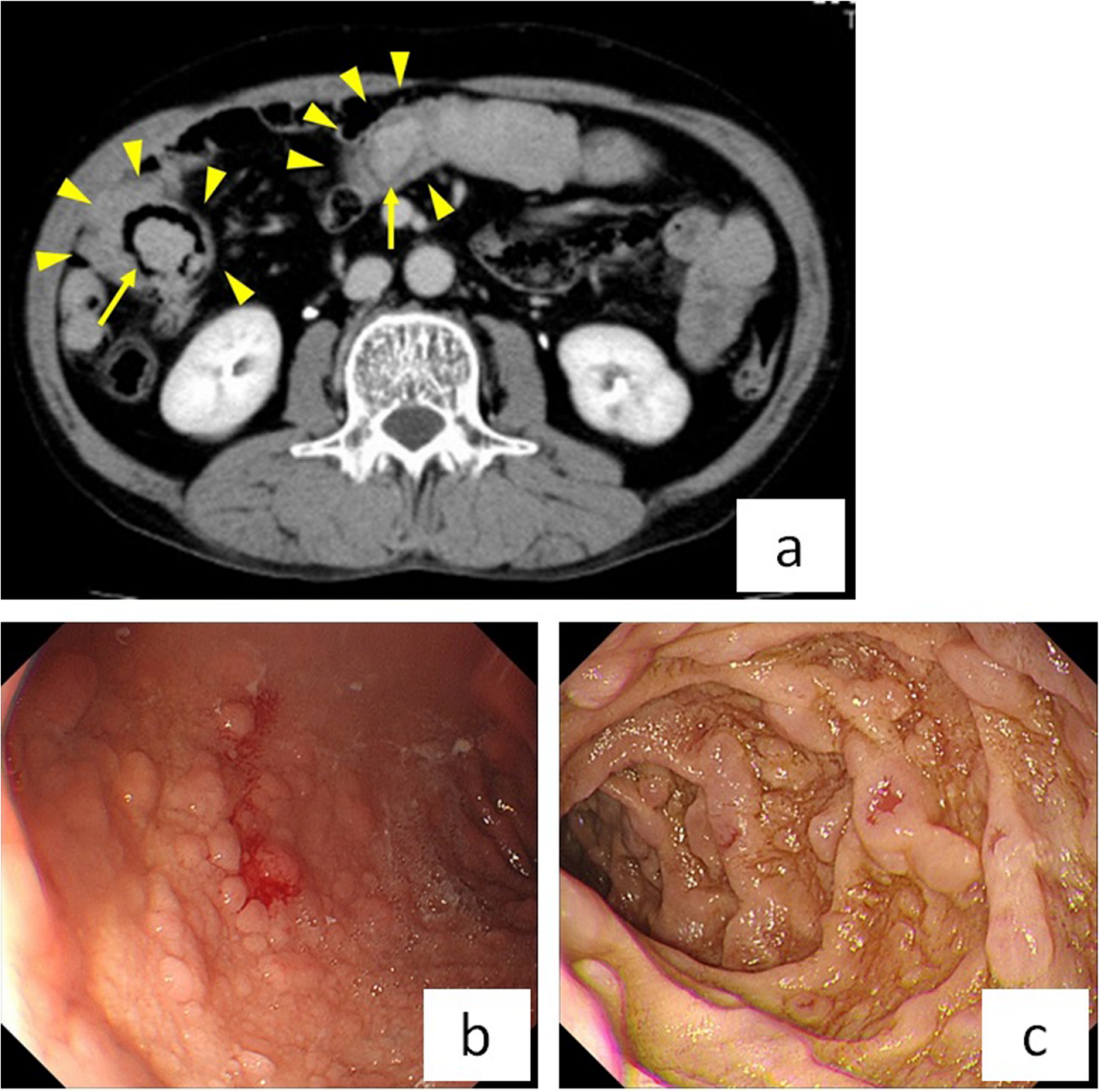
**a** In the small intestine, CT shows multiple intussusceptions (arrowheads) and polyps (arrows). **b**, **c** Gastrointestinal fiberscopy shows multiple polyposis in the stomach **b** and duodenum **c**

**Fig. 2:**
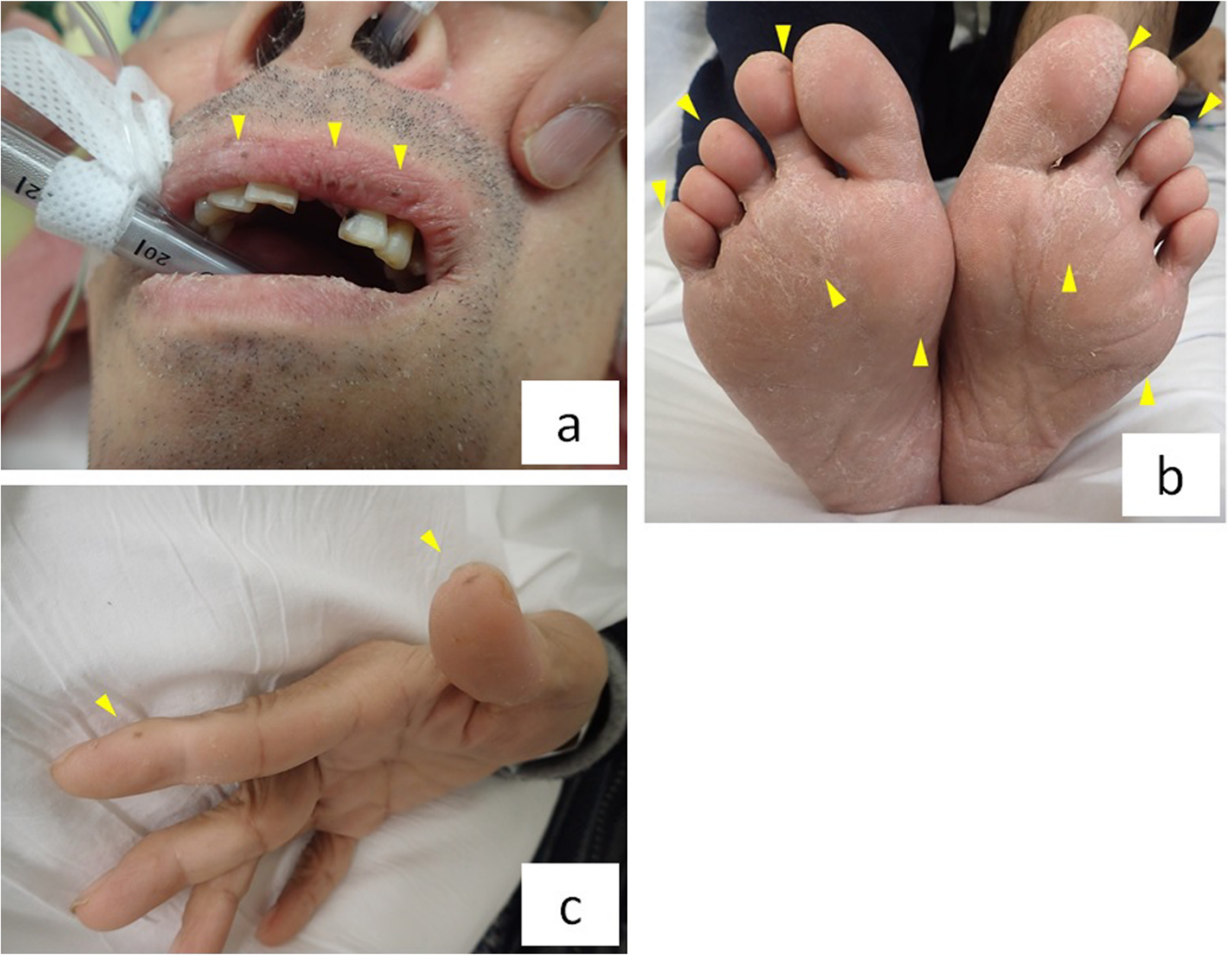
**a**–**c** The patient demonstrated pigmentation of the lips, fingers, and toes (arrowheads)

A 62-year-old male had a history of hypertension, hyperlipidemia, hyperuricemia, benign prostatic hyperplasia, and hepatic dysfunction. He was hospitalized for treatment of deep vein thrombosis (DVT) and pulmonary embolism (PE) with heparin and factor Xa inhibitor. Magnetic resonance imaging showed that DVT extended from both the left external iliac vein and the right femoral vein to the periphery (Supplementary Fig. 1a). PE involved an artery in the right lower lung, and echocardiography showed no pulmonary hypertension (Supplementary Fig. 1b). DVT and PE were caused by protein C deficiency type 2. Since the patient had progressive anemia (hemoglobin 11.1 to 8.0 g/dl for 11 days) and fecal occult blood testing was positive, gastrointestinal hemorrhage was suspected. The results of the hematological examination are shown in Supplementary Table 1. Although he had mild constipation about every 3 days, he was able to eat and had no abdominal pain. In the small intestine, computed tomography (CT) showed multiple intussusceptions and polyps (Fig. 1a). Upper gastrointestinal fiberscopy demonstrated multiple polyposis of the stomach and duodenum (Fig. 1b). The polyps were not actively bleeding, but bled easily with contact. On physical examination, the patient had numerous areas of pigmentation on his lips, fingers, and toes (Fig. 2a-c). Based on the above findings, he was diagnosed with PJS. Since the small intestinal polyps were thought to have caused gastrointestinal bleeding and multiple small intestinal intussusceptions that increase the risk of future bowel obstruction, we decided to perform laparoscopic disinvagination of the small intestine.

### Surgical procedure

A small incision (3 cm) was made at the navel and a wound retractor was placed. The first port was inserted through the wound retractor. Four additional ports were inserted as follows: at the wound retractor, in the left upper quadrant, and in the right and left lower quadrants.

**Fig. 3:**
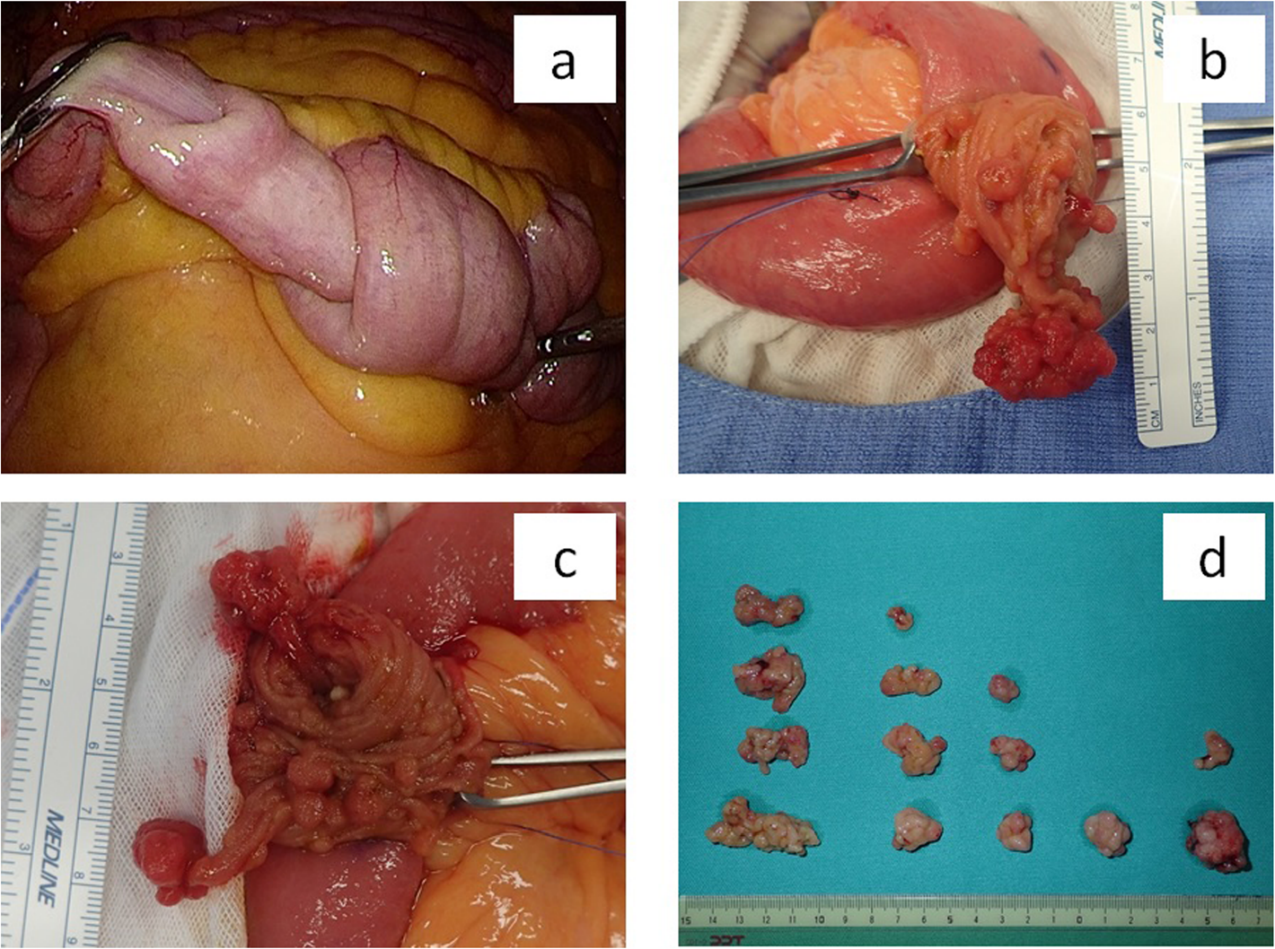
**a** Laparoscopic surgery reveals small intestinal intussusceptions. **b**, **c** There are multiple long pedunculated polyps in the intestine. **d** Fourteen long, pedunculated polyps were resected. Each had a minimum dimension of over 1 cm, and the largest had a maximum dimension of 4 cm

We examined the entire small intestine laparoscopically and identified five intussusceptions, located 110, 140, 180, 200, and 225 cm from the ligament of Treitz (Fig. 3a). After marking the intussusceptions with sutures, we removed the small intestine from the abdomen and created 2- to 3-cm longitudinal incisions at the five locations, then removed as many polyps as possible since they were risk factors for intussusception (Fig. 3b, c). Fourteen polyps were resected; all were pedunculated and had a minimum dimension of at least 1 cm, and the largest had a maximum dimension of 4 cm (Fig. 3d).

### Clinical outcomes

The patient resumed oral ingestion of water on postoperative day (POD) 1, liquid food on POD5, and porridge on POD6. He received a transfusion of red cell concentrate (280 ml) on POD2 since his hemoglobin was 6.9 g/dl. There were no complications and he was discharged from the hospital on POD9.

### Pathological findings

**Fig. 4:**
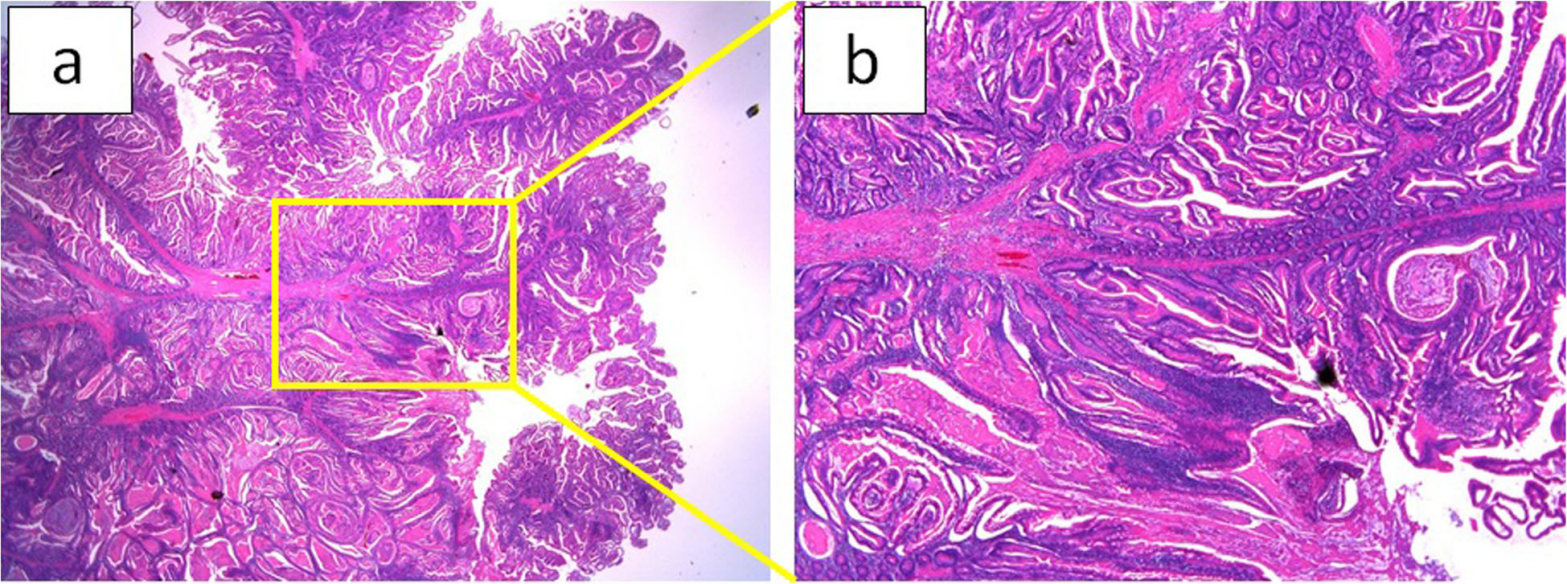
**a**, **b** The pathological diagnosis was hamartomatous polyps

All polyps were pathologically diagnosed as hamartomatous polyps compatible with PJS (Fig. 4a, b).

## Discussion and conclusions

It has been reported that patients with PJS have a high risk of malignant tumors of the gastrointestinal tract, pancreas, lung, uterus, ovary, breast, and testis due to germline mutations in *STK11*, which is a tumor suppressor gene [7, 10]. Boardman et al. reported that the relative risks of gastrointestinal, gynecological, and breast cancer were 50.3, 20.3, and 15, respectively [11].

Polyps in PJS may be found throughout the gastrointestinal tract, excluding the esophagus, and are most common in the small intestine [12]. Since polyps can prolapse and also cause intussusception, intestinal obstruction, and bleeding, PJS patients often need to undergo surgery for these conditions until 20 years of age [13]. Spiegelman et al. reported that the mean age at primary surgery was 15.0 (2–39) years and the average number of surgeries per patient was 2.4 [14].

Although PJS polyps are considered to be hamartomas, some studies have shown that they may have an adenomatous component [15, 16]. A hamartoma–adenoma–carcinoma pathway was therefore proposed as a pathological mechanism [17]. Ohmiya et al. reported that 1.3% of small intestinal polyps smaller than 20 mm consisted partially of adenoma, and none of these was malignant [16]. However, among those larger than 21 mm, 30% had an adenomatous component and 3.3% were malignant. In particular, those larger than 15 mm sometimes harbored an adenomatous component and caused invagination. The authors recommended that whenever possible, polyps larger than 10 mm should be resected in order to lengthen the interval to the next polypectomy. Therefore, since a relatively high proportion of polyps larger than 10 mm contain adenomatous tissue, it is recommended that these be resected [16].

Double-balloon enteroscopy (DBE) and capsule endoscopy devices are able to directly visualize the inside of the small intestine, and DBE can also be used to resect polyps. Reported complications of DBE include bleeding (1.4–2.7%), perforation (1.4–2.9%), and acute pancreatitis (2.7%) [18–20]. If patients have previously undergone laparotomy, DBE examination is more difficult due to the presence of adhesions [16]. Therefore, intraoperative enteroscopy (IOE) is recommended in patients with PJS who have a history of laparotomy since polyps can be precisely located and removed, and be available if there is perforation of the small intestine.

Surgery for small intestinal intussusception requires not only intestinal repositioning but also resection of polyps, since these are a risk factor for multiple conditions. If an irreversible disorder is present, especially intestinal necrosis, bowel resection is necessary. However, multiple enterotomies should be avoided whenever possible, as these may result in short bowel syndrome. In the present case, since IOE was not available at our hospital, we made a small incision near each small intestinal intussusception and successfully resected the nearby polyps without the need for bowel resection. Since all of the polyps with a dimension of over 1 cm were pedunculated and were obviously located within the mucosa on both visual inspection and palpation during surgery, we decided to resect the nearby polyps without bowel resection. Pathological findings revealed that all polyps were benign tumors situated within the mucosa. Moreover, since laparoscopic-assisted resection is a minimally invasive operation, we were able to minimize the patient’s suffering.

After surgery, the digestive tract should be evaluated by CT and/or gastrointestinal tract examination to identify sizeable gastroenterological polyps that may cause intussusception or bleeding. Moreover, patients with PJS are at increased risk of cancers of various organs, including the esophagus, stomach, small bowel, colon, pancreas, lung, testis, breast, uterus, ovary, and cervix [21, 22]. However, there is no consensus about which of these organs should be monitored, or with what frequency [1]. We recommend performing CT and a complete gastrointestinal tract examination every 1–2 years. If necessary, patients with PJS should be referred to a breast oncologist, gynecologist, or urologist.

## Supplementary Information

**Additional file 1: Figure S1** a. Magnetic resonance imaging reveals DVT extending from both the left external iliac vein and right femoral vein to the periphery. Figure S1b. Echocardiography reveals PE involving an artery of the right lower lung, with no pulmonary hypertension (arrowhead).

**Additional file 2: Table S1**. Results of hematological examination.

## Data Availability

The material supporting the conclusion of this review has been included within the article.

## Abbreviations

PJS: Peutz-Jeghers syndrome
*STK11*: Serine–threonine kinase 11 gene
CT: Computed tomography
DVT: Deep vein thrombosis
PE: Pulmonary embolism
POD: Postoperative day
DBE: Double-balloon enteroscopy
IOE: Intraoperative enteroscopy
