# Supplementary figures and images for: Laparoscopic-assisted disinvagination and polypectomy for multiple intussusceptions induced by small intestinal polyps in patients with Peutz-Jeghers syndrome: a case report
Source: World J Surg Oncol. 2021 Jan 21;19:22. doi: 10.1186/s12957-021-02133-5 (PMC7819471; doi:10.1186/s12957-021-02133-5)

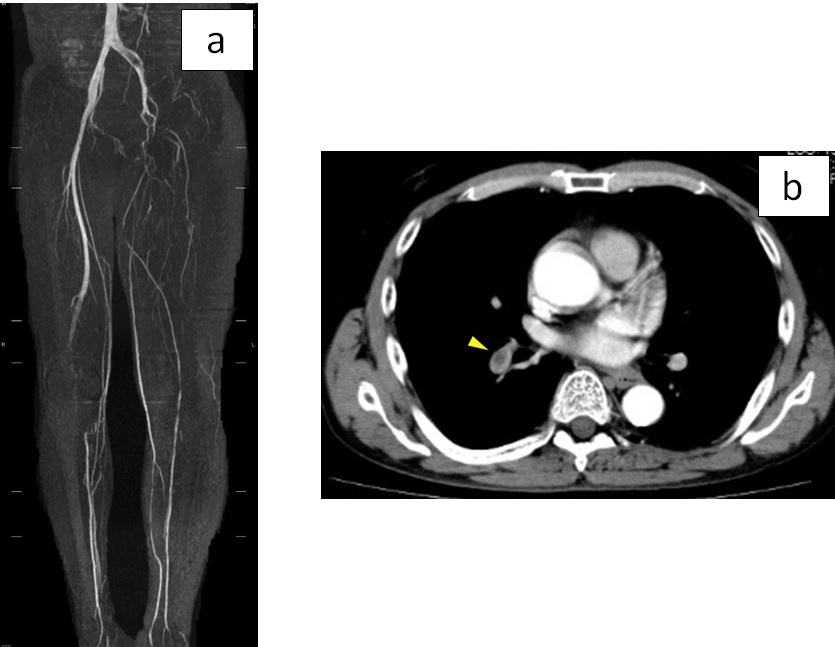

Supplement: Supplementary file 1 — Additional file 1: Figure S1 a. Magnetic resonance imaging reveals DVT extending from both the left external iliac vein and right femoral vein to the periphery. Figure S1b. Echocardiography reveals PE involving an artery of the right lower lung, with no pulmonary hypertension (arrowhead). [file 12957_2021_2133_MOESM1_ESM.jpg]
