# Supplementary material for: Laparoscopic-assisted disinvagination and polypectomy for multiple intussusceptions induced by small intestinal polyps in patients with Peutz-Jeghers syndrome: a case report
Source: World J Surg Oncol. 2021 Jan 21;19:22. doi: 10.1186/s12957-021-02133-5 (PMC7819471; doi:10.1186/s12957-021-02133-5)
Supplement: Supplementary file 2 — Additional file 2: Table S1. Results of hematological examination. [file 12957_2021_2133_MOESM2_ESM.docx]

**Supplementary Table 1.** **Results of hematological examination**

| Parameters | Value | Units |
| --- | --- | --- |
| White blood cells | 33 | ×10^2^/μl |
| Red blood cells | 255 | ×10^4^/μl |
| Hemoglobin | 8.0 | g/dl |
| Hematocrit | 24.1 | % |
| Platelets | 39.5 | ×10^4^/μl |
| Albumin | 3.2 | g/dl |
| Total bilirubin | 0.3 | mg/dl |
| Aspartate aminotransferase | 52 | IU/L |
| Alanine aminotransferase | 69 | IU/L |
| Alkaline phosphatase | 105 | IU/L |
| Lactase dehydrogenase | 170 | IU/L |
| Gamma-glutamyl transpeptidase | 102 | IU/L |
| Blood urea nitrogen | 14.4 | mg/dl |
| Creatinine | 0.99 | mg/dl |
| Sodium | 141 | mEq/l |
| Potassium | 4.7 | mEq/l |
| Chloride | 106 | mEq/l |
| C-reactive protein | 0.04 | mg/dl |
| Carcinoembryonic antigen | 1.6 | ng/ml |
| Carbohydrate antigen 19-9 | 2.0 | U/ml |
| Prothrombin time-international normalized ratio | 0.91 |  |
| Activated partial thromboplastin time | 27 | sec |
| Fibrinogen | 277 | mg/dl |
| Antithrombin III | 95.9 | % |
| D-dimer | 5.0 | µg/ml |
| Fibrin and fibrinogen degradation product | 7.5 | µg/ml |
| Protein C antigen | 137 | % |
| Protein C activity | 46 | % |
| Protein S antigen | 80 | % |
| Protein S activity | 85 | % |
